# Clinical features and survival of pregnancy-associated breast cancer: a retrospective study of 203 cases in China

**DOI:** 10.1186/s12885-020-06724-5

**Published:** 2020-03-23

**Authors:** Bo-yue Han, Xiao-guang Li, Hai-yun Zhao, Xin Hu, Hong Ling

**Affiliations:** 1 Department of Breast Surgery, Fudan University Shanghai Cancer Center, Fudan University, 270 Dong-an Rd, Shanghai, 200032 China; 2 Department of Oncology, Shanghai Medical College, Fudan University, Shanghai, 200032 China; 3 Department of Breast Surgery, Key Laboratory of Breast Cancer in Shanghai, Fudan University Shanghai Cancer Center, Fudan University, Shanghai, 200032 China

**Keywords:** Pregnancy-associated breast cancer, First-pregnancy, Non-first-pregnancy, Lactation, Survival

## Abstract

**Background:**

Pregnancy-associated breast cancer (PABC) is an aggressive disease, and since Chinese authority began to encourage childbearing in 2015, the incidence of PABC has increased. This study investigated the characteristics and survival of PABC patients.

**Methods:**

Patients with PABC who underwent surgery at Fudan University, Shanghai Cancer Center between 2005 and 2018 were enrolled. Data concerning the tumor characteristics, maternal state (whether first or non-first pregnancy) and survival outcome were recorded. Pearson Chi-square tests were used to compare the characteristics of the tumors, and Kaplan-Meier methods were used to perform the survival analysis.

**Results:**

Overall, 203 PABC patients were recruited. Since 2015, 65.5% of non-first pregnant women were diagnosed with breast cancer, it’s 5.7 fold of the incidence of PABC in non-first pregnant women. No significant differences in tumor characteristics were observed between the patients who were in their first pregnancy and those in non-first pregnancy. Among the entire PABC population, luminal B breast cancer accounted for the largest proportion (38.4%), followed by triple-negative breast cancer (TNBC, 30.0%). The distribution of the molecular subtypes of PABC and non-PABC differed (*P* < 0.001) as follows: in the PABC patients, Luminal B 38.4%, Triple negative breast cancer (TNBC) 30.1%, Human Epidermal Growth Factor Receptor 2 (HER-2) overexpression 15.8%, and Luminal A 10.8%; in the non-PABC patients, Luminal A 50.9%, Luminal B 20.1%, TNBC 17.4%, and HER-2 overexpression 8.0%. The 3-year disease free survival (DFS) of all PABC patients was 80.3%. The 3-year DFS of the patients in the first-pregnancy group was 78.4%, and that of the patients in the non-first-pregnancy group was 83.7% (*P* = 0.325).

**Conclusions:**

Our study proved that the proportion of women who developed PABC during the second or third pregnancy was extremely high relative to the newborn populations. The patients in the PABC population tended to present more luminal B and TNBC breast cancer than the non-PABC patients.

## Background

Breast cancer is the most common cancer among women [1]. Pregnancy-associated breast cancer (PABC) is defined as breast cancer diagnosed during pregnancy or within 1 year after pregnancy [2]. PABC is a very rare type of cancer. The incidence of PABC reportedly ranges from 0.2 to 3.8% [3, 4].

In October 2015, Chinese authority abolished the restriction in which a couple can have only one child to actively address the aging of the population. Subsequently, we observed a sharp increase in PABC at our center, and non-first pregnancies accounted for a large proportion, which attracted our attention. However, a thorough understanding of this problem is lacking; thus, we performed an investigation of PABC in the Chinese population. We enrolled 203 women treated at Fudan University, Shanghai Cancer Center (FUSCC) to study the clinical characteristics and prognosis of PABC patients.

## Patients and methods

### Participant eligibility

In this retrospective study, we reviewed the medical records of patients who underwent surgery between January 2005 and December 2018 at the Department of Breast Surgery, FUSCC. The eligible patients included women who had regional invasive unilateral breast cancer, with their first symptoms occurring during pregnancy or lactation. The lactation period usually refers to the first year after childbirth. Patients diagnosed with stage IV breast cancer or previously diagnosed breast cancer, ductal or lobular atypical hyperplasia, sarcomas or phyllodes tumors were excluded from our study (Fig. S1). We also enrolled women who were diagnosed with breast cancer at FUSCC during the same period to compare the molecular subtypes (*n* = 43,721). This retrospective study was approved by the Ethics Committee Review Board of FUSCC (050432).

### Data collection

All patients diagnosed with PABC between January 2005 and December 2018 were enrolled in this study. To analyze the clinicopathological characteristics of PABC patients, the study variables included the age of the patients, gestational period at the appearance of the first symptoms (months), family history of breast cancer, surgery type and other treatments (adjuvant/neoadjuvant chemotherapy, radiotherapy, endocrine therapy and target therapy), pathologic tumor size, lymph node status, histological grade, estrogen receptor (ER) and progesterone receptor (PR) status, expression of human epidermal growth factor receptor-2 (HER-2), expression of Ki-67, etc. A status of either ER or PR positive was defined as hormone receptor (HR) positive.

The data of all recruited patients were collected for the PABC characteristic analysis. For the survival analysis, patients diagnosed with PABC after 2016 were excluded to ensure a follow-up time longer than 3 years. Disease-free survival (DFS) was defined as the time between the first date of diagnosis to any locoregional recurrence, including ipsilateral breast, local/regional lymph nodes of the disease, any contralateral breast cancer, any distant metastasis of the disease, or any secondary malignancy, whichever occurred first [5, 6].

### Statistical analysis

Pearson Chi-square tests were used to compare the histopathological characteristics of the tumors and clinical features of the patients among the different subgroups. The Kaplan-Meier methods were used to perform the survival analysis. All tests were two-sided, and a *P*-value less than 0.05 was considered statistically significant. All statistical analyses were performed using SPSS statistical software version 25.0 package (IBM Corporation, Armonk, NY, USA).

## Results

### General information

In total, 203 patients were diagnosed with PABC between 2005 and 2018 in FUSCC, and the median age of the study population was 33 years (range, 23 years to 46 years). The population was divided into the first-pregnancy group, which included women with breast cancer during the pregnancy or lactation period of their first child, and the non-first-pregnancy group, which included women with PABC during the pregnancy or lactation period of their second, third or greater child. Among the patients, 79 (38.9%) women developed breast cancer during their first pregnancy period (first-pregnancy group), and 124 (61.1%) women were assigned to the non-first-pregnancy group. Since 2015, 65.5% of non-first pregnant women were diagnosed with breast cancer, while only 25% of newborns were non-first births in Shanghai (according to the China Health and Wellness Development Statistics). Thus, the incidence of PABC among non-first pregnancy women was 5.7-fold higher than that among first-pregnancy women.

### Tumor characteristics

Table 1 shows the distribution of the tumor characteristics according to the first/non-first pregnancy subgroups. The first-pregnancy group was younger than the non-first-pregnancy group (*P* < 0.01). The proportion of HR-positive tumors in the first-pregnancy group was 57.0%, while the proportion in the non-first-pregnancy group was 47.6% (*P* = 0.281). In the first-pregnancy group, the proportion of HER-2-positive tumors was 26.6%, while that in the non-first-pregnancy group was 36.3% (*P* = 0.108).

**Table 1:** Patient characteristics and tumor characteristics according to first and non-first pregnancy subgroup

Endocrine therapy					0.281
Yes	45	57.0	59	47.6	
No	34	43.0	61	49.2	
Target therapy					0.757
Yes	17	21.5	29	23.4	
No	62	78.5	95	76.6	

*Abbreviations*: *HR* Hormone receptor, *HER-2* Human epidermal growth factor receptor-2, *IDC* Invasive ductal carcinoma, *DCIS* Ductal carcinoma in situ, *ILC* Invasive lobular carcinoma, *SLNB* Sentinel lymph node biopsy, *ALND* Axillary lymph node dissection, *pCR* Pathological complete remission

(a): HR positive: ER (estrogen receptor) positive or/and PR (progesterone receptor) positive

(b): Pearson Chi-square tests between first pregnancy group and non-first pregnancy group

Among all patients, 23 (11.3%) patients chose to terminate their pregnancies and receive immediate treatment (abortion group), 66 (32.5%) patients were diagnosed with PABC during pregnancy and chose to delay treatment until the fetus was born (non-abortion group), and the remaining 114 (56.2%) PABC cases were diagnosed during the lactation period (lactation group) (Table S1).

### Molecular subtypes

**Fig. 1:**
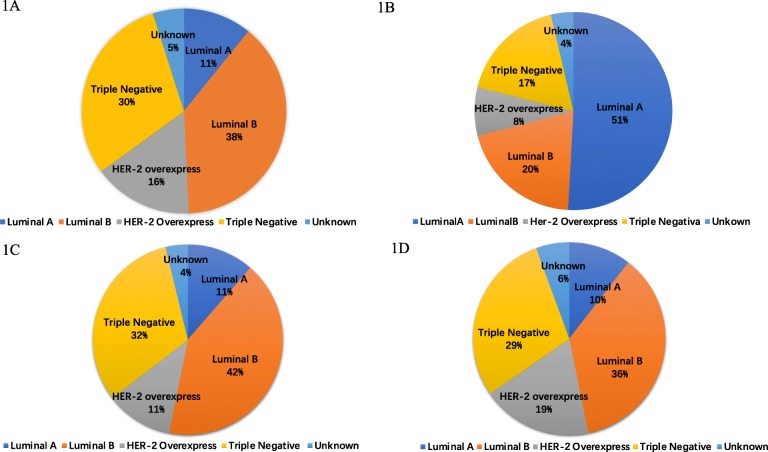
Molecular subtypes of the PABC, breast cancer other than PABC, PABC developed in women’s first pregnancy and non-first pregnancy. **a** Molecular subtypes of the PABC, *n* = 203. **b** Molecular subtypes of breast cancer other than PABC (non-PABC), *n* = 43,721. **c** Molecular subtypes of the PABC developed in women’s first pregnancy (First-Pregnancy subgroup), *n* = 79. **d** Molecular subtypes of the PABC not developed in women’s first pregnancy (Non-First-Pregnancy subgroup), *n* = 124. The *P* value was less than 0.001, by using Pearson Chi-square tests to compare the distribution of molecular subtypes in PABC patients (**a**) and non-PABC patients (**b**), demonstrating a difference. The P value was 0.554, by using Pearson Chi-square tests to compare the distribution of molecular subtypes in First-pregnancy group (**c**) and Non-first-pregnancy group (**d**), demonstrating no statistical significance. PABC=Pregnancy-associated breast cancer; ER = Estrogen Receptor; PR = Progesterone Receptor; HER-2 = Human Epidermal Growth Factor Receptor-2, HR (Hormone Receptor) +: Either ER or PR+. Luminal A: ER+, PR+, HER-2 (−), Ki-67 < 14%; Luminal B: HR+, Ki-67 ≥ 14%; HR+, HER-2(+); ER+, PR-; Her-2 overexpression: HR (−), HER-2 (+); TNBC (Triple negative breast cancer): ER (−), PR (−), HER-2 (−)

Among the entire PABC population, luminal B breast cancer accounted for the largest proportion (38.4%), followed by triple-negative breast cancer (TNBC, 30.0%). Compared with PABC, the non-PABC patients showed a significant distribution of molecular subgroups as follows: luminal A breast cancer was the most common (50.9% in non-PABC vs. 10.8% in PABC, *P* < 0.001), followed by luminal B breast cancer (20.1% in non-PABC vs. 38.4% in PABC, *P* < 0.001). The proportion of both TNBC and HER-2 overexpression breast cancer was much smaller in the non-PABC patients (17.4% in non-PABC vs. 30.1% in PABC, *P* < 0.001; 8.0% in non-PABC vs. 15.8% in PABC, P < 0.001, respectively) (Fig. 1). It was demonstrated that a greater proportion of patients with PABC had the luminal B and TNBC types of cancer. A trend similar to that observed in the total PABC population was observed in both the first-pregnancy group and non-first pregnancy group (Fig. 1).

### Treatments

Compared with the non-first-pregnancy group, the first-pregnancy group preferred to delay treatment until the fetus was born (proportion of non-abortion cases: 85.7% vs. 68.9%, *P* = 0.092). The times from initial symptoms to initiation of treatment in the first-pregnancy and non-first-pregnancy groups were 6.20 months and 4.67 months, respectively (*P* = 0.106).

In total, 196 (96.6%) women received adjuvant/neoadjuvant chemotherapy, and anthracycline combined taxane chemotherapy (53.5%) was the most commonly used regimen. Among the patients, 84 patients received neoadjuvant chemotherapy, and 18 (21.4%) patients achieved a pathologic complete response (pCR). Although trastuzumab was recommended for all patients with HER-2 overexpression tumors, not all patients could afford the high cost. Among the patients with HER-2 overexpression tumors, 46 (69.7%) patients received trastuzumab as the target therapy (Table S1).

### Survival analysis

**Fig. 2:**
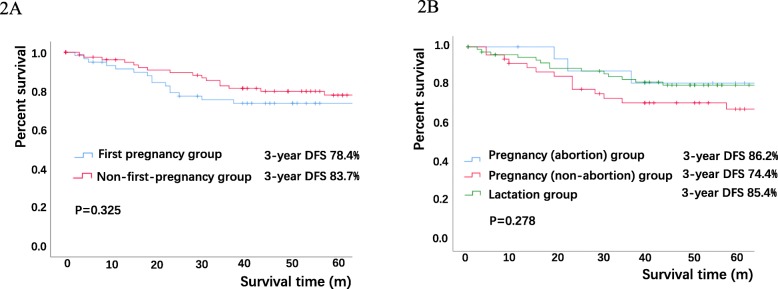
Survival of PABC patients in different subgroups. **a** Comparison of 3-year DFS of patients with PABC developed in their first pregnancy (first pregnancy group) and PABC developed in non-first pregnancy. **b** Survival curve of patients with PABC developed in pregnancy phase and underwent abortion (abortion subgroup), in pregnancy phase but no abortion (non-abortion group) and PABC developed in lactation phase. The 3-year DFS was estimated between First-pregnancy group and Non-first-pregnancy group by Log-rank test with a P value of 0.325. The 3-year DFS was estimated among among Pregnancy (non-abortion) subgroup, Pregnancy (abortion) subgroup and Lactation subgroup of PABC by Log-rank test with a P value of 0.278. PABC=Pregnancy Associated Breast Cancer; DFS = Disease Free Survival

Among all patients diagnosed with PABC before 2016, the median follow-up period was 59.0 months (range, 2 months to 144 months). The 3-year disease free survival (DFS) of all PABC patients was 80.3%, the DFS of the patients in the first-pregnancy group was 78.4%, and the DFS of the patients in non-first-pregnancy group was 83.7% (*P* = 0.325, Fig. 2a). The 3-year DFS in the pregnancy (abortion) group, pregnancy (non-abortion) group and lactation group was 86.2, 74.4 and 85.4%, respectively (*P* = 0.278, Fig. 2b).

## Discussion

We reviewed 25 studies conducted over the past 20 years to gain a deeper understanding of PABC (Table 2). The incidence of PABC reportedly ranges from 0.2–3.8% [3, 4, 6]. PABC used to be a rare disease in China. However, recently, the number of cases increased. In our study, we observed that the frequency of PABC in non-first pregnancy women has increased as women started to have second children since Chinese authority abolished the restriction that couples could only have one child. Our study found that the proportion of PABC developed in non-first pregnancy women was 5.7-fold higher than that developed in first-pregnancy women. We reviewed the literature and found a study conducted in Taiwan that enrolled 26 PABC patients, and most patients (*n* = 18) were first-pregnancy women [30]. These inconsistent results may be due to the small enrollment number. As the largest breast center in East China, our center has treated more than 6000 primary breast cancer patients per year, ensuring less bias in our study. Other than the above-mentioned study, we found no other studies mentioning the difference in the incidence of PABC between first-pregnancy women and non-first pregnancy women.

**Table 2:** Literature review of pregnancy-associated breast cancer since 2000

Author	References	Year	Mean Age	PABC	Breast Cancer During Preganncy	Breast Cancer Postpartum	Definition of Postpartum (Year After Pregnancy)	Non-PABC^(a)^	Follow-Up (years)	Conclusion
Ibrahim	[7]	2000	34	72	72		Unspecfied	216	4	No differfence in OS
Aziz	[8]	2003	32	24			1	48	7	No differfence in OS
Beadle	[9]	2009	33	104			1	548	10	No differfence in OS,LRR,DM
Murphy	[10]	2012	35	99			1	186	18	No differfence in OS
Framarino-DeiMalatesta	[11]	2014	37.2	22	22		Unspecfied	45	10	No differfence in OS
Baulies	[12]	2015		56			Unspecfied	73	5	No differfence in DFS
Genin	[13]	2015	35	87			1	174	9	No differfence in OS,DFS
Boudy	[14]	2018	35	49	49		Unspecfied	104	3.3	No difference in OS, DFS, BCSS
Rodriguez	[15]	2008	< 55	797			1	4177	13	Worse OS for PABC
Moreira	[16]	2010	35	87			1	252	10	Worse OS for PABC
Johansson	[17]	2011	< 45	1110			2	14,611	15	Worse OS or PABC
Ali	[18]	2012	33	40			1	40	10	Worse OS and DFS for PABC
Dimitrakakis	[19]	2013	34.3	39			1	39	5	Worse OS for PABC
Madaras	[20]	2014	34	31			1	31	10	Worse OS and DFS for PABC
Sánchez	[21]	2014	35.3	15			1	251	10	Worse OS for PABC
Kim	[22]	2017	33.7	344			1	668	10	Worse OS for PABC
Suleman	[23]	2018	34	110	110		Unspecfied	114	2.8	Worse DFS for PABC
Bae	[24]	2018		411			1	83,381		Worse OS for PABC
Bae	[25]	2018	33.5	40			1	2770	4.4	Worse BCSS and DFS for PABC
Johansson	[26]	2018		778			2	1661	10	Worse OS for PABC
Daling	[27]	2002	< 45	83		83	2	309	5	Worse OS for BC Postpartum
Mathelin	[28]	2008	33.8	40	18	22	1	61	10	OS,DFS: BCP worse than BC Postpartum than Non-PABC
Halaska	[29]	2009	33.7	32	16	16	1	32	10	DFS: BC Postpartum worse than BCP and Non-PABC
Johansson	[17]	2013	< 44	323	45	278	2	3915	9	DFS: BC Postpartum worse than BCP than Non-PABC
Yang	[30]	2014	34	26	15	11	1	104	5	OS: BC Postpartum worse than BCP and Non-PABC

*Abbreviations*: *OS* Overall survival, *LRR* Local recurrence, *DM* Distant metastasis, *DFS* Disease free survival, *BCSS* Breast cancer specific survival, *BC* Breast cancer, *BCP* Breast cancer during pregnancy, *PABC* Pregnancy associated breast cancer

(a): The non-pregnancy-associated breast cancer patients recruited as control groups in the studies

In our study, we observed a significant difference in the molecular subtypes between the PABC and non-PABC cases. Luminal B breast cancer accounted for the largest proportion of all PABC patients, followed by triple-negative breast cancer. Consistent with our study, Soo reported that luminal B breast cancer (43.6%) and TNBC (35.9%) predominated in PABC [24]; while one study presented a different conclusion and showed that TNBC ranked first (48.4%) [20]. Some studies did not list the molecular types but reported the HR and HER-2 status and demonstrated that PABC was more prone to be HR-negative tumors, but no difference in the HER-2 status was reported compared with non-PABC as follows: HR negative (50.0% in PABC vs. 36.1% in non-PABC, *P* < 0.001 (Yun et al.)) [25], HR negative (32.6% in PABC vs. 15.9% in non-PABC, *P* = 0.014 (Jessica et al.)) [22], and HR negative (59.4% in PABC vs. 34.4% in non-PABC, *P* = 0.03 (Michael et al.)) [31]; only one study reported by Soo showed a higher HER-2 positive rate in PABC patients as follows: HER-2 positive (38.5% in PABC vs. 19.2% in non-PABC, *P* = 0.006) [29]. Although these views vary, all studies indicated that PABC tended to present with more aggressive tumors.

The 3-year disease free survival (DFS) of all PABC patients at FUSCC was 80.3%. We reviewed the literature, and the survival of PABC patients reportedly fluctuates over a large range. Wagner reported a very low survival as follows: 5-year overall survival (OS) of 29.7% and 10-year OS of 19.2% among PABC patients [28]; however, Carole showed that the 5-year OS was 87.5% and that the 10-year OS was 70.0% [29]. The survival rates of the PABC patients compared to those of the non-PABC patients were conflicting. Most studies [15, 16, 18–21, 23–26, 28, 32] demonstrated a worse prognosis in PABC after excluding prognostic factors, including age, the tumor size, and lymph node status, while eight studies [7–14] showed no difference in survival between PABC and non-PABC patients after correcting for these factors.

Our analysis showed that the Kaplan-Meier survival curve of the first-pregnancy group was below that of the non-first-pregnancy group. However, there was no statistically significant difference. We speculate that the two groups might have survival differences, but these differences are unclear in this study due to the rare incidence and limited case number. We have no supporter. We will collect more cases to make it clear in 10 years.

Five studies classified PABC into antepartum and postpartum breast cancer, and three studies showed that the prognosis of PABC occurring postpartum was worse than that of PABC occurring during gestation [17, 30, 31]; Mathelin concluded that the prognosis of PABC occurring during the antepartum period was worse [29]; and Daling indicated that PABC occurring postpartum had a worse survival rate than non-PABC [27]. The survival analysis in our study showed no difference. In our study, we found that patients in early pregnancy were more likely to terminate their pregnancies, while those in late pregnancy usually preferred to delay treatment until the delivery of the fetus.

Starting chemotherapy in mid-late pregnancy without delaying chemotherapy until after delivery is generally preferred as unnecessary delays may result in a worse prognosis. FAC (fluorouracil, adriamycin and cyclophosphamide) is a commonly used chemotherapy regimen that has been shown to be safe in mid-late pregnancy [33]. Doxorubicin and cyclophosphamide can be excreted through milk and, therefore, are prohibited during lactation [33]. However, in China, people generally do not undergo chemotherapy during mid-late pregnancy. Mid-pregnancy women with PABC choose to either terminate the pregnancy or delay chemotherapy until delivery, while late-pregnancy women usually start chemotherapy treatment after delivery. In our study population, 20 (30.3%) PABC patients with HER-2 positivity did not receive Herceptin treatment, including 18 (85.7%) patients who were diagnosed with PABC before 2017. In China, Herceptin was not included in the scope of medical insurance reimbursement until 2017.

It should be acknowledged that there were some limitations in our present study. This study was a single-center study. The follow-up of the patients in the non-first-pregnancy group was short because the restriction was abolished in 2015. We could only obtain the 3-year DFS data. Moreover, some tumor characteristics were absent. The HER-2 status of 9 people was unknown probably because the patients refused to undergo further FISH analyses due to the high cost at that time.

## Conclusions

In conclusion, our study proved that the incidence of PABC developed during the second or third pregnancy was higher than that developed in women’s first pregnancy. The patients in the PABC population tended to present more luminal B and TNBC breast cancers than the non-PABC patients. Our single-center study provides some information regarding the characteristics and survival rates of PABC patients. However, further research investigating PABC in a large population and investigations of the physiological mechanisms is needed in the future.

## Supplementary information

**Additional file 1: Figure S1.** Flow chart of patient selection. FUSCC=Fudan University Shanghai Cancer Center; PABC=Pregnancy-associated breast cancer.

**Additional file 2: Figure S2.** Molecular subtypes of the Pregnancy (non-abortion), Pregnancy (abortion) and Lactation subgroup of PABC. S2 A: Molecular subtypes of the Pregnancy (non-abortion) subgroup, *n* = 66. S2 B: Molecular subtypes of the Pregnancy (abortion) subgroup, *n* = 23. S2 C: Molecular subtypes of the Pregnancy Lactation subgroup, *n* = 114. The *P* value was 0.551, by using Pearson Chi-square tests to compare the distribution of molecular subtypes in the Pregnancy (non-abortion) (S2 A), Pregnancy (abortion) (S2 A) and Lactation subgroup (S2 A) of PABC. PABC=Pregnancy-associated breast cancer; ER = Estrogen Receptor; PR = Progesterone Receptor; HER-2 = Human Epidermal Growth Factor Receptor-2, HR (Hormone Receptor) (+): Either ER or PR (+). Luminal A: ER (+), PR (+), HER-2 (−), Ki-67 < 14%; Luminal B: HR (+), Ki-67 ≥ 14%; HR (+), HER-2 (+); ER (+), PR (−); Her-2 overexpression: HR-,HER-2 (+); TNBC (Triple negative breast cancer): ER (−), PR (−), HER-2 (−)

**Additional file 3: Table S1.** Patient characteristics and tumor characteristics according to pregnancy (abortion), pregnancy (non-abortion) and lactation subgroup. (a): HR positive: ER (estrogen receptor) positive or/and PR (progesterone receptor) positive. (b): Pearson Chi-square tests between pregnancy (non-abortion) group and pregnancy (abortion) group (c): Pearson Chi-square tests between pregnancy (non-abortion) group and lactation group.

## Data Availability

Not applicable.

## Abbreviations

PABC: Pregnancy-associated breast cancer
TNBC: Triple-negative breast cancer
HER-2: Human Epidermal Growth Factor Receptor 2
DFS: Disease free survival
FUSCC: Fudan University, Shanghai Cancer Center
ER: Estrogen receptor
PR: Progesterone receptor
HR: Hormone receptor
pCR: Pathologic complete response
FAC: Fluorouracil, adriamycin and cyclophosphamide
