# Supplementary figures and images for: Clinical features and survival of pregnancy-associated breast cancer: a retrospective study of 203 cases in China
Source: BMC Cancer. 2020 Mar 23;20:244. doi: 10.1186/s12885-020-06724-5 (PMC7092544; doi:10.1186/s12885-020-06724-5)

Fig. S1

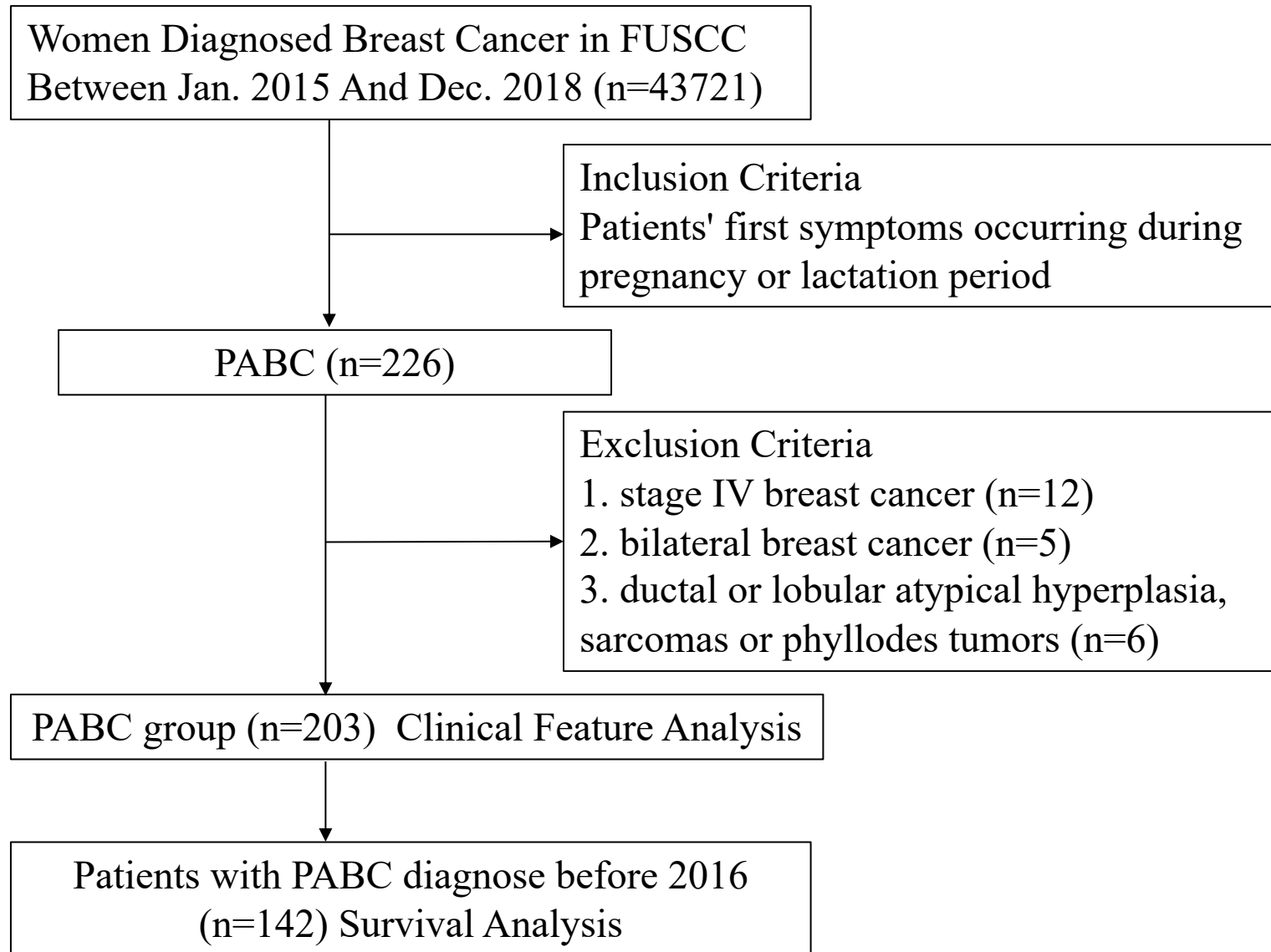

Supplement: Supplementary file 1 — Additional file 1: Figure S1. Flow chart of patient selection. FUSCC=Fudan University Shanghai Cancer Center; PABC=Pregnancy-associated breast cancer. [file 12885_2020_6724_MOESM1_ESM.pdf]

Fig. S2A

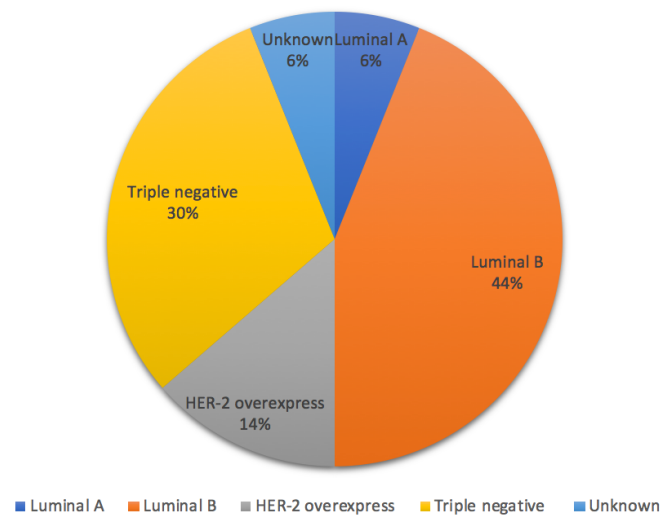

Fig. S2B

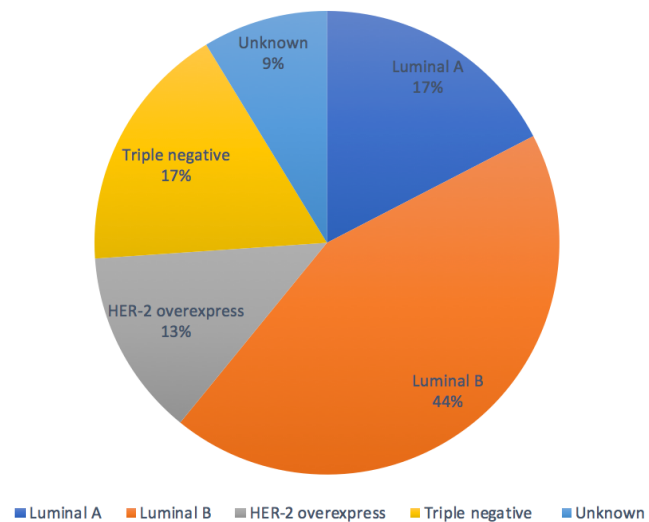

Fig. S2C

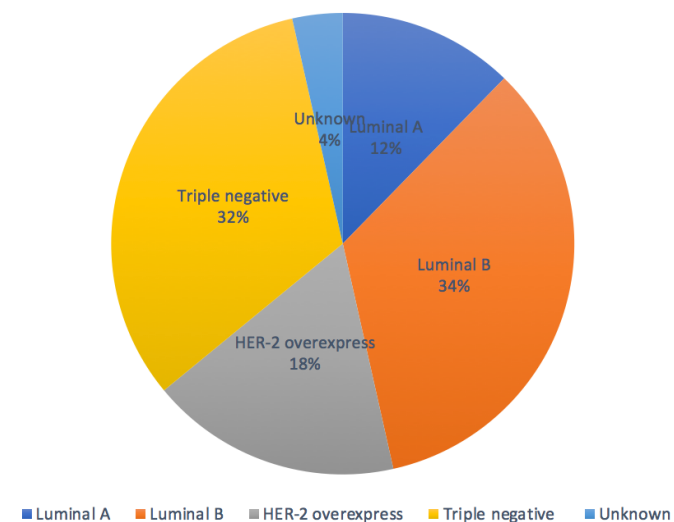

Supplement: Supplementary file 2 — Additional file 2: Figure S2. Molecular subtypes of the Pregnancy (non-abortion), Pregnancy (abortion) and Lactation subgroup of PABC. S2 A: Molecular subtypes of the Pregnancy (non-abortion) subgroup, n = 66. S2 B: Molecular subtypes of the Pregnancy (abortion) subgroup, n = 23. S2 C: Molecular subtypes of the Pregnancy Lactation subgroup, n = 114. The P value was 0.551, by using Pearson Chi-square tests to compare the distribution of molecular subtypes in the Pregnancy (non-abortion) (S2 A), Pregnancy (abortion) (S2 A) and Lactation subgroup (S2 A) of PABC. PABC=Pregnancy-associated breast cancer; ER = Estrogen Receptor; PR = Progesterone Receptor; HER-2 = Human Epidermal Growth Factor Receptor-2, HR (Hormone Receptor) (+): Either ER or PR (+). Luminal A: ER (+), PR (+), HER-2 (−), Ki-67 < 14%; Luminal B: HR (+), Ki-67 ≥ 14%; HR (+), HER-2 (+); ER (+), PR (−); Her-2 overexpression: HR-,HER-2 (+); TNBC (Triple negative breast cancer): ER (−), PR (−), HER-2 (−) [file 12885_2020_6724_MOESM2_ESM.pdf]
